# Supplementary material for: Integrative omics analysis of the termite gut system adaptation to Miscanthus diet identifies lignocellulose degradation enzymes
Source: Commun Biol. 2020 Jun 1;3:275. doi: 10.1038/s42003-020-1004-3 (PMC7264248; doi:10.1038/s42003-020-1004-3)
Supplement: Supplementary file 2 — Description of Additional Supplementary Files [file 42003_2020_1004_MOESM2_ESM.pdf]

Descriptions of Additional Supplementary Files

Supplementary Data 1: Details of the 16S rRNA gene amplicon high-throughput sequencing. Data is normalised to 10,000 reads per sample. Technical replicates are indicated with the same sample name.

Supplementary Data 2: Linear discriminant analysis (LDA) effect size of bacterial OTUs significantly enriched in control microbiome (bacterial population in a termite gut feeding natural food) and under Miscanthus diet (bacterial population in a termite gut feeding on Miscanthus spp. grass).

Supplementary Data 3: Relative abundance of genes (metagenomics) and gene transcripts (metatranscriptomics, host transcriptomics) assigned to Clusters of Orthologous Groups (COGs) for gut microbiome and host transcriptome.

Supplementary Data 4: KEGG ontology profiles (KOs) assigned to genes (de novo MG) and gene transcripts (de novo MT) of prokaryotic (gut microbiome) and eukaryotic (termite host) origin.

Supplementary Data 5: Linear discriminant analysis (LDA) effect size of KOs significantly enriched in the two main bacterial populations, Fibrobacteres and Spirochaetae, based on the RNA-seq results. Metabolic modules (only complete and with one block missing) reconstruction for the two main bacterial populations in the termite gut, Spirochaetae and Fibrobacteres, based on the RNA-seq results.

Supplementary Data 6: Characterisation of heterologously produced CAZymes.

Supplementary Data 7: Characteristics of the reconstructed metagenome assembled genomes (MAGs) based on the checkM evaluation. Putative CAZymes and CAZymes genes clusters on the de novo reconstructed MG contigs.

Supplementary Data 8: Transporters reconstruction, comparison of the de novo metagenomics, de novo metatranscriptomics and RNA-seq for the termite gut microbiome.

Supplementary Data 9: Comparison of CAZy genomic content (number of genes) between the different termite species with sequenced genomes.

Supplementary Data 10: Per phylum summary of the sub-cellular localisation of GH-assigned genes and further functionally annotated with HotPep.
