# Supplementary material for: Integrative omics analysis of the termite gut system adaptation to Miscanthus diet identifies lignocellulose degradation enzymes
Source: Commun Biol. 2020 Jun 1;3:275. doi: 10.1038/s42003-020-1004-3 (PMC7264248; doi:10.1038/s42003-020-1004-3)
Supplement: Supplementary file 13 — Reporting Summary [file 42003_2020_1004_MOESM13_ESM.pdf]

nature research

Corresponding author(s): Magdalena Calusisnka

Last updated by author(s): 29/04/2020

Reporting Summary

Nature Research wishes to improve the reproducibility of the work that we publish. This form provides structure for consistency and transparency in reporting. For further information on Nature Research policies, see Authors & Referees and the Editorial Policy Checklist.

Statistics

For all statistical analyses, confirm that the following items are present in the figure legend, table legend, main text, or Methods section.

n/a	Confirmed	
☐	☒	The exact sample size (*n*) for each experimental group/condition, given as a discrete number and unit of measurement
☐	☒	A statement on whether measurements were taken from distinct samples or whether the same sample was measured repeatedly
☒	☐	The statistical test(s) used AND whether they are one- or two-sided<br>*Only common tests should be described solely by name; describe more complex techniques in the Methods section.*
☒	☐	A description of all covariates tested
☒	☐	A description of any assumptions or corrections, such as tests of normality and adjustment for multiple comparisons
☐	☒	A full description of the statistical parameters including central tendency (e.g. means) or other basic estimates (e.g. regression coefficient) AND variation (e.g. standard deviation) or associated estimates of uncertainty (e.g. confidence intervals)
☒	☐	For null hypothesis testing, the test statistic (e.g. *F*, *t*, *r*) with confidence intervals, effect sizes, degrees of freedom and *P* value noted<br>*Give P values as exact values whenever suitable.*
☒	☐	For Bayesian analysis, information on the choice of priors and Markov chain Monte Carlo settings
☒	☐	For hierarchical and complex designs, identification of the appropriate level for tests and full reporting of outcomes
☐	☒	Estimates of effect sizes (e.g. Cohen's *d*, Pearson's *r*), indicating how they were calculated

Our web collection on statistics for biologists contains articles on many of the points above.

Software and code

Policy information about availability of computer code

Data collection	MiSeq software v2.6<br>NextSeq System Suite v2.1.3
Data analysis	Usearch v.7.0.1090_win64<br>mothur 1.36.1<br>R 3.6.1<br>LEfSe https://huttenhower.sph.harvard.edu/galaxy/<br>CLC Genomics Workbench 9.5.2<br>MyCC https://sourceforge.net/projects/sb2nhri/files/MyCC/<br>metaWRAP 1.1.3<br>checkM 1.0.12<br>PhyloPhlan 0.99<br>FastANI 1.2<br>blastn 2.5.0+<br>blastp 2.5.0+<br>MEGA X<br>SortMeRNA 2.0<br>BUSCO 2.0<br>dbCAN-fam-HMMs.txt.v6<br>hotpep https://omictools.com/hotpep-tool<br>LipoP version 1.0

For manuscripts utilizing custom algorithms or software that are central to the research but not yet described in published literature, software must be made available to editors/reviewers. We strongly encourage code deposition in a community repository (e.g. GitHub). See the Nature Research guidelines for submitting code & software for further information.

Data

Policy information about availability of data

All manuscripts must include a data availability statement. This statement should provide the following information, where applicable:

- Accession codes, unique identifiers, or web links for publicly available datasets
- A list of figures that have associated raw data
- A description of any restrictions on data availability

The raw sequencing data used in this study was deposited in SRA database under following project numbers: PRJNA587606 (16S rRNA amplicon sequencing), PRJNA587423 (Metagenomics) and PRJNA587406 (Metatranscriptomics). OTU sequences were deposited in GenBank under project numbers PRJNA586754 and PRJNA434195. COII nucleotide sequences are available in GenBank under accession numbers MN803317-19. MG and MT assemblies and all other data underlying the findings of this study are available from the corresponding author upon reasonable request.

Field-specific reporting

Please select the one below that is the best fit for your research. If you are not sure, read the appropriate sections before making your selection.

☐ Life sciences ☐ Behavioural & social sciences ☒ Ecological, evolutionary & environmental sciences

For a reference copy of the document with all sections, see nature.com/documents/nr-reporting-summary-flat.pdf

Ecological, evolutionary & environmental sciences study design

All studies must disclose on these points even when the disclosure is negative.

Study description	In this study, we investigated the dynamic adaptation of a Cortaritermes spp. higher termite gut lignocellulolytic system to degrade Miscanthus biomass under the controlled laboratory conditions. Approach-wise, our experimental design resembles the commonly used enrichment strategy, where a nature-derived microbial inoculum is grown in liquid batch cultures and progressively changes in composition to yield a consortium specialised in degradation of a specific biomass. As lignocellulose degradation by the termite gut system results from the synergistic cooperation between the host and its gut microbiome, here we investigated the lignocellulolytic potential of Cortaritermes spp. at the holobiont level, using an integrative omics approach (gene amplicon study, metagenomics and metatranscriptomics) combined with the biochemical characterisation of selected bacterial carbohydrate active enzymes.
Research sample	The research samples consisted of three termite nests of Nasutitermitinae, that were collected in French Guiana (in proximity to Sinnamary town, radius of 5 km to GPS: N 05°24.195’ W 053°07.664’). Termite nests were transported to the laboratory where colonies were maintained in separate glass containers at 26 °C, 12h light and 12h dark, and 90 % humidity conditions. Termite colonies were fed with Miscanthus grass winter harvest rich in recalcitrant lignocellulose, for a period of up to nine months (collaboration with the University Paris 13). The selected termite species were identified as feeding on grass in their natural environment. Termite species were identified by morphology and by sequencing of the partial COII marker gene.
Sampling strategy	Mature worker-caste individuals were sampled in regular monthly time intervals before (sample taken before Miscanthus diet corresponds to “wild-microbiome”) and following Miscanthus diet (samples correspond to “Miscanthus-adapted microbiome”). Termite specimens were cold immobilized, surface-cleaned with 80 % ethanol and 1 x PBS and decapitated. Hindgut compartments of termite guts were dissected (n ≈ 30 per replicate, minimum three replicates per sample) and preserved directly in liquid nitrogen. Additionally, for a sample selected for metagenomics analysis (LM1 time point 8 months; LM1_8) the hindgut luminal fluid was collected. Samples were stored at -80 °C until further processing. Total DNA and RNA were co-extracted from all samples using the AllPrep PowerViral DNA/RNA Kit (Qiagen) following manufacturer’s protocol.
Data collection	Extracted DNAs and RNAs, following adequate library preparation steps, were sequenced in a high throughput manner using either the Illumina MiSeq (Luxembourg Institute of Science and Technology) or Illumina NextSeq (University of Luxembourg).
Timing and spatial scale	Termite gut samples were collected in regular monthly time intervals, over the period of nine months, starting in January 2017.
Data exclusions	One termite colony LM1_2 did not adapt to the laboratory conditions and died after few months, thus it was excluded from further analysis.
Reproducibility	Two termites colonies were fed with Miscanthus diet and when we studied the evolution of their gut microbiomes, we could notice the enrichment of similar bacterial OTUs, that would indicate towards the reproducibility of our results between biological replicates. The amplicon sequencing study included triplicate samples. Technical replicates were not included in the metatranscriptomics studies (due to the increased cost), however similar trends in terms of the gene expression profiles were observed between the two studies samples representing the Miscanthus-adapted microbiome group, and they were different from the control sample. Moreover, in our previous study (under revision in another journal), we have shown high reproducibility between metatranscriptomics technical replicates. Therefore, we assume that our results are reproducible. For the enzymatic study, each time a triplicate sample was included.
Randomization	There were two groups of samples, (1) before the application of a Miscanthus diet(control samples corresponding to sampling points LM_1 and LM3_1) and (2) Miscanthus-adapted termite gut system, corresponding to all the other samples.
Blinding	Blinding was not relevant in our study.

Did the study involve field work? ☒ Yes ☐ No

Field work, collection and transport

Field conditions	The field work was carried out near Sinnamary, French Guiana. The temperature was around 30°C and the relative humidity was around 75%. The rainfall was quite common at this time of the year.
Location	The termite colonies were collected in a savannah area near Sinnamary (radius of 5 km to GPS: N 05°24.195' W 053°07.664') at 0.5 meters above sea level.
Access and import/export	No important efforts were made to access to the place of collection. The termite colonies were in the savannah so we drove off-road for a short time and walked in the savannah up to the nests. Termite colonies were stored into a plastic container with an access to distilled water and were transported to Paris, France (autorization number TREL1820249A/108 issued by the French Ministery of Ecological and Solidarity Transition to David Sillam-Dussès, French associate professor at the University Paris 13).
Disturbance	No specific disturbance

Reporting for specific materials, systems and methods

We require information from authors about some types of materials, experimental systems and methods used in many studies. Here, indicate whether each material, system or method listed is relevant to your study. If you are not sure if a list item applies to your research, read the appropriate section before selecting a response.

Materials & experimental systems

n/a	Involved in the study	
☒	☐	Antibodies
☒	☐	Eukaryotic cell lines
☒	☐	Palaeontology
☐	☒	Animals and other organisms
☒	☐	Human research participants
☒	☐	Clinical data

Methods

n/a	Involved in the study	
☒	☐	ChIP-seq
☒	☐	Flow cytometry
☒	☐	MRI-based neuroimaging

Animals and other organisms

Policy information about studies involving animals; ARRIVE guidelines recommended for reporting animal research

Laboratory animals	The study did not involve laboratory animals.
Wild animals	Three colonies of grass-feeding higher termites from the sub-family Nasutitermitinae, Cortaritermes intermedius (not pest species and not endangered species) were collected in French Guiana. Each colony contained one queen, one king and several thousands of sterile workers and soldiers. The colonies were taken from the soil where they were built, stored into a ventilated plastic container with an access to distilled water and were transported to Paris, France.
Field-collected samples	The colonies were maintained in separate glass containers at 26 °C, 12h light and 12h dark, and 90 % humidity conditions. When needed, termite specimens were cold immobilized, surface-cleaned with 80 % ethanol and 1 x PBS and decapitated for experimental purposes. The colonies are still alive in the breeding room.
Ethics oversight	No ethical approval was required (insects are not subjected to ethical approval).

Note that full information on the approval of the study protocol must also be provided in the manuscript.
